# Developmentally Regulated Sesquiterpene Production Confers Resistance to *Colletotrichum gloeosporioides* in Ripe Pepper Fruits

**DOI:** 10.1371/journal.pone.0109453

**Published:** 2014-10-06

**Authors:** Sangkyu Park, Ae Ran Park, Soonduk Im, Yun-Jeong Han, Sungbeom Lee, Kyoungwhan Back, Jeong-Il Kim, Young Soon Kim

**Affiliations:** 1 Department of Biotechnology, Chonnam National University, Gwangju, Korea; 2 Kumho Life Science Laboratory, Chonnam National University, Gwangju, Korea; 3 Research Division for Biotechnology, Advanced Radiation Technology Institute, Jeongeup, Korea; Institute of Genetics and Developmental Biology, Chinese Academy of Sciences, China

## Abstract

Sesquiterpenoid capsidiol, exhibiting antifungal activity against pathogenic fungus, is accumulated in infected ripe pepper fruits. In this study, we found a negative relation between the capsidiol level and lesion size in fruits infected with *Colletotrichum gloeosporioides*, depending on the stage of ripening. To understand the developmental regulation of capsidiol biosynthesis, fungal-induced gene expressions in the isoprenoid biosynthetic pathways were examined in unripe and ripe pepper fruits. The sterol biosynthetic pathway was almost shut down in healthy ripe fruits, showing very low expression of hydroxymethyl glutaryl CoA reductase (*HMGR*) and squalene synthase (*SS*) genes. In contrast, genes in the carotenoid pathway were highly expressed in ripe fruits. In the sesquiterpene pathway, 5-epi-aristolochene synthase (*EAS*), belonging to a sesquiterpene cyclase (*STC*) family, was significantly induced in the ripe fruits upon fungal infection. Immunoblot and enzyme activity analyses showed that the STCs were induced both in the infected unripe and ripe fruits, while capsidiol was synthesized discriminatively in the ripe fruits, implying diverse enzymatic specificity of multiple STCs. Thereby, to divert sterol biosynthesis into sesquiterpene production, infected fruits were pretreated with an SS inhibitor, zaragozic acid (ZA), resulting in increased levels of capsidiol by more than 2-fold in the ripe fruits, with concurrent reduction of phytosterols. Taken together, the present results suggest that the enhanced expression and activity of EAS in the ripe fruits play an important role in capsidiol production, contributing to the incompatibility between the anthracnose fungus and the ripe pepper fruits.

## Introduction

Plants induce multiple arrays of defense systems upon pathogen attack. Among these, chemical defense involves the production of substances that are potentially toxic to the pathogen, known as phytoalexins [1]. Capsidiol has been found to be the major phytoalexin produced by inoculation of pepper (*Capsicum annuum*) and tobacco (*Nicotiana tabacum*) with pathogens [2]–[4]. Capsidiol production generally occurs in or around the infection site of the pathogen, and plays a defensive role against pathogen manifestation. In pepper, various organs are known to be capable of producing capsidiol [5]–[7], which inhibits the growth of pathogenic fungi and bacteria [8], [9]. Even though ripe fruits of other plants are generally susceptible to pathogen infection [10], pepper fruits revealed distinct responses to the virulent anthracnose fungus, *Colletotrichum gloeosporioides*, showing an incompatible interaction with the ripe red fruits [11]. Thus, it would be interesting to determine whether capsidiol is involved in this defense reaction of the ripe pepper fruits against the anthracnose fungus.

Capsidiol, a bicyclic dihydroxylated sesquiterpene, is derived from the isoprenoid pathway in the cytoplasm [12]. The majority of the genes encoding the enzymes of the isoprenoid pathway have been characterized in fungi, animals and plants. However, isoprenoid biosynthesis appears to be more complex in higher plants, which generate thousands of natural isoprenoid products [13], [14]. The biosynthetic pathway begins with the conversion of acetyl-CoA to the five-carbon isopentenyl diphosphate (IPP) through the cytosolic mevalonate (MVA) pathway. Hydroxymethyl glutaryl CoA reductase (HMGR), which catalyzes the formation of mevalonate, is known as a rate-limiting step in the isoprenoid pathway [15]. In this pathway, IPP and dimethylallyl diphosphate (DMAPP) are condensed to form geranyl diphosphate, which then reacts with another IPP molecule to yield farnesyl diphosphate (FPP). Squalene synthase (SS) then catalyzes the condensation of two FPP molecules to form squalene, which is the first enzymatic step in sterol synthesis.

In plants, FPP is the common precursor for the biosynthesis of sequiterpenes as well as sterols. The synthesis of sequiterpenes is initiated by cyclization of all-trans FPP into 5-epi-aristolochene (EA), a reaction catalyzed by a sesquiterpene cyclase (STC) known as EA synthase (EAS). Capsidiol, a dihydroxy-5-epi-aristolochene, is then synthesized by the reaction with 5-epi-aristolochene hydroxylase (EAH) [16]. Neither EAS nor EAH is constitutively expressed in shoots of plants, but they are both induced upon treatment with elicitors [17]–[19]. To date, four different cDNA sequences for *STC* have been reported in *Capsicum annuum*, and a UV-inducible *STC*, SC1, has been demonstrated to have EAS enzyme activity [20]. The existence of multiple STCs suggests that the respective enzymes may participate in specific reactions for the cyclization of FPP molecules into various products. On the other hand, it has been reported that the induction of EAS was accompanied by the suppression of SS in plant cell cultures [21], [22]. Such alterations presumably trigger the diversion of carbon flow from the central isoprenoid pathway into the sesquiterpene synthesis pathway [23].

In plastids, the methylerythritol phosphate (MEP) pathway is responsible for the synthesis of IPP, providing the precursor for the synthesis of carotenoids. Channeling of two IPP pools between the cytosolic and plastidial pathway of isoprenoid biosynthesis has been observed in plant cells [24], [25]. In addition, direct evidence of crosstalk between the two IPP biosynthetic pathways has been provided in snapdragon flowers, occurring uni-directionally from the plastids to the cytoplasm [26]. Since carotenoid biosynthesis is triggered developmentally in ripening pepper fruits, two independent pathways for IPP biosynthesis take place concurrently, in both the cytosol and the plastid of the ripe red fruits. Therefore, it should be considered whether plastid IPP may contribute to the synthesis of sesquiterpene in the cytoplasm of infected ripe pepper fruits.

In this study, to gain an understanding of the regulatory mechanism controlling the biosynthesis of capsidiol, fungal-induced expression of the genes involved in the isoprenoid pathways were investigated in pepper fruits. The expression patterns of the genes in the squalene, sesquiterpene and carotenoid biosynthetic pathways were compared between susceptible unripe and resistant ripe fruits, after infection with anthracnose fungus. Moreover, quantitative assays of capsidiol and phytosterols were conducted in the infected fruits. Here, we show that capsidiol is primarily biosynthesized during the incompatible interaction of the ripe fruits with the anthracnose fungus. Furthermore, we demonstrated that the amount of capsidiol produced can be increased with concurrent reduction of phytosterols by inhibiting the squalene synthase, but only in ripe fruits.

## Materials and Methods

### Plant materials

Commercial pepper used in the study (*Capsicum annuum* L. cv. Nockkwang) was obtained from Heungnong Seed Co. Ltd., Korea. Plants were grown at 25°C under greenhouse conditions. Fully-grown unripe green and ripe red fruits from three-month-old pepper plants were used.

### Fungal pathogen and inoculation

Inoculum preparation and artificial inoculation procedures were performed as previously described [27]. Briefly, the KG13 isolate of *C. gloeosporioides* was grown on potato dextrose agar (Sigma) at 28°C. Seven-day-old colonies were then flooded with distilled water and gently scraped from the plates, after which the inoculum concentration was adjusted to 5×10^5^ mL^-1^. Next, 10 µL of the conidial suspension was drop-inoculated at two sites on the surface of the detached pepper fruits. The inoculated fruits were then placed in an acrylic box that was moistened and sealed tightly to maintain relative humidity near 100%, after which they were incubated at 25°C. For analysis, a piece (7×7 mm) of pericarp was taken from the inoculated sites of the fruits at 1, 2, 3, and 4 days. In addition, the development of anthracnose symptoms was monitored until 9 days after infection. To examine the effect of the inhibitor of squalene synthase on the infected fruits, 10 µM zaragozic acid (ZA; Sigma) was applied directly to the surface of the fruits for 3 h at 28°C before inoculation with fungal spores separately next to the ZA drop on the fruits.

### Antifungal activity of capsidiol

Capsidiol was extracted from pepper fruits treated with 0.1% cellulase for 2 days, and purified as described previously [28]. The antifungal activity of capsidiol was analyzed by investigating the germination and colony growth of the anthracnose fungus, *C. gloeosporioides.* Spore germination was monitored by microscopic examination in a cover glass inoculated with 500 spores in sterile distilled water containing 10 µL of 0.02–1.0 mM of capsidiol. Spores were incubated for 24 hours at 28°C, and then counted for the germination and appressorium formation in at least three microscopic fields.

The disc diffusion method was used to evaluate the inhibition of colony growth on PDA media, as described in a previous report [29]. Briefly, mycelial discs with a diameter of 5 mm were taken from 7 day- old cultures grown on PDA. The discs were then inoculated in the center of a fresh PDA plate. When the diameter of the fungal growth reached approximately 3 cm, the filter discs (Whatman No. 1, Ф 6 mm) were moistened with 10 µL of 0.02, 0.2 and 1 mM capsidiol, or a sterile water control, and placed equidistantly on the plates. Squalene (Sigma) and farnesol (Aldrich) at 1 mM concentration were also used as controls. Three replicates were performed for each treatment, and the plates were incubated for 3 days at 28°C.

### Expression analysis of isoprenoid pathway genes by Northern blots

The primers used for the cloning of isoprenoid pathway genes are shown in Table S1. Twenty nanogram aliquots of total RNA isolated from the inoculated red fruits were used as the template for reverse transcription, performed with the ImProm-II Reverse Transcription System (Promega). The nucleotide sequences of the genes in the isoprenoid pathway were determined and compared using a BLAST search (NCBI database). For Northern blot analysis, RNA was extracted from pepper fruits using an RNeasy Plant Kit (Qiagen). Total RNA (10 µg/lane) was separated on 1.2% agarose gels, and then blotted onto a Hybond N^+^ membrane (GE Healthcare). The blots were then prehybridized at 65°C for 2 h, after which hybridization was performed overnight at 65°C with [α-^32^P] dCTP-labeled cDNA probes in prehybridization solution. Radiolabeled probes were prepared using a random primer-labeling kit (GE Healthcare).

### Isolation of soluble and microsomal proteins

Samples were homogenized in an extraction buffer (50 mM Tris, pH 8.0, 2 mM EDTA, 2 mM DTT, 0.25 M sucrose) at 4°C, then subjected to centrifugation at 8,000 *g* for 15 min. The supernatant was then used as the total soluble protein for EAS enzyme assays. For microsomal preparation, the supernatant was centrifuged at 100,000 *g* for 60 min. The microsomal pellet was then resuspended in 200 µL of buffer (20 mM Tris·HCl, pH 7.5, 10 mM MgCl_2_ and 2 mM 2-mercaptoethanol), and the extracts were used for SS enzyme assays. Protein concentrations were determined using a protein assay dye (Bio-Rad) with bovine serum albumin as the standard.

### Western blot analysis and enzyme activity assays for SS and EAS

For immunoblot analysis, anti-SS serum was raised against a KLH-conjugated peptide corresponding to residues 367–380 of pepper squalene synthase (Accession number, AF124842.1). Proteins were separated by 12% SDS-PAGE, then electro-transferred onto polyvinylidene fluoride (PVDF) membranes. Membranes were then exposed to polyclonal anti-EAS mouse [20] and anti-SS rat antibodies. A western blotting system (GE Healthcare) was employed to visualize the bands using a 1∶10,000 dilution of HRP-coupled anti-mouse or rat antibody.

For enzyme activity assays, the conversion of [^3^H]-FPP into cyclic products was used to measure the activity of EAS, according to the method described previously [20]. The enzyme activity of SS was determined by measuring the conversion of [^3^H]-FPP into squalene, according to the method described previously [21].

### Expression analysis of STC genes

RT-PCR analyses were carried out to examine the expression levels of four *STC* genes, including *SC1* (AF061285), *SC2* (AF212433), *PSC1* (AF326118) and *PSC2* (AF326117). RNA samples were isolated from infected unripe and ripe fruits at 0, 24, 48, and 72 HAI, respectively. Primer sequences used for the RT-PCR analyses are shown in Table S2. To quantitate the RT-PCR results, real time RT-PCR of fungal-induced *SC1* and *SC2* were performed with the Brilliant III Ultra Fast SYBR Green QPCR Master Mix (Agilent Technologies) using Stratagene MX3000P with a software program (MxPro-Mx3000P v.4.10Build 389, Schema85). The ubiquitin-conjugating protein gene (*UBI-3*) was used as a reference gene for normalization [30].

### Extraction and quantification of capsidiol and sterols

Capsidiol was extracted using a mixture of chloroform and methanol (2∶1, v/v), then quantified using a gas chromatograph (Varian Cp-3800, USA) equipped with an FID 40 flame ionization detector and an RTX1 capillary column. The column was programmed as follows: initial temperature of 100°C, followed by an increase to 300°C over 20 min (10°C/min), where the temperature was maintained for 5 min. The injector and detector were maintained at 180°C and 300°C, respectively. Nitrogen was applied as the carrier gas at a flow rate of 1 mL/min. Sterols were extracted from 20 mg of freeze-dried pepper fruits using the method described in a previous report [31]. GC analyses were then conducted under a source temperature of 250°C, an injection temperature of 250°C, and a column temperature program: 80°C for 1 min, followed by an increase to 280°C at a rate of 20°C/min, where the temperature was maintained for 8 min. He was used as the carrier gas in the GC analyses, applied at a flow rate 1 mL/min. The chemical identity of capsidiol was confirmed with comparison to the published retention index and mass spectrum.

### Statistical Analysis

All experimental data were subjected to the Analysis of Variance test (ANOVA) using IBM SPSS statistics 20 software. Significant differences in the mean values from different treatments were determined using the least significant difference (LSD) and DMRT methods at *P*<0.05. All data were expressed as the mean ± SD of at least three independent replicates.

## Results

### Accumulation of capsidiol in the infected ripe red fruits of pepper

**Figure 1:**
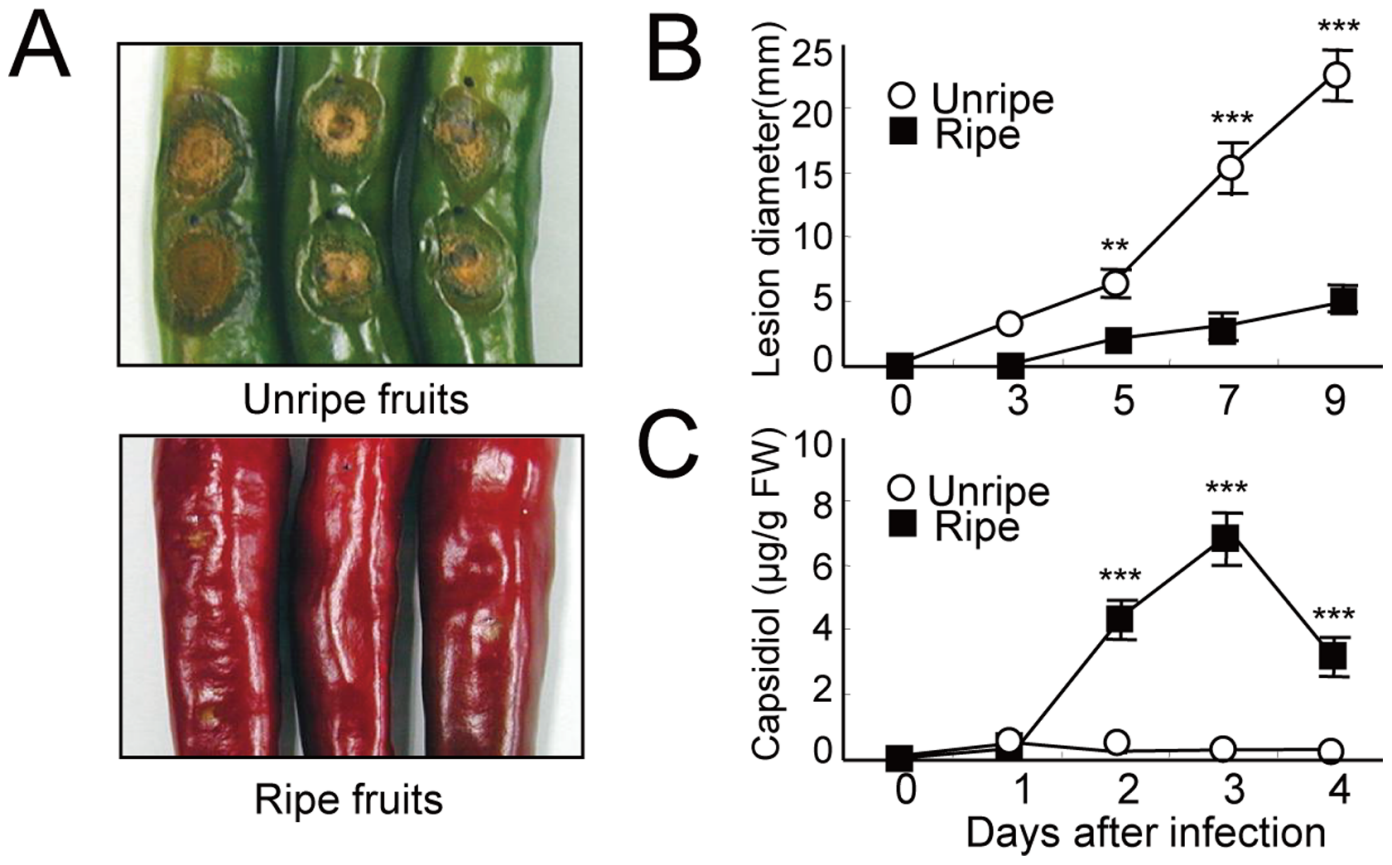
Development of anthracnose symptoms in infected pepper fruits and antifungal activity of capsidiol. **A**, Representative anthracnose symptoms on unripe green and ripe red fruits 9 days after infection. **B**–**C**, Measurements of lesion diameters (B) and capsidiol levels (C) in the unripe and ripe fruits infected with *C. gloeosporioides*. Statistically significant changes between unripe and ripe fruits are indicated by ** and ***, indicating *P*<0.01 and 0.001, respectively, using two-way ANOVA. At least 20 fruits were counted per replicate. Each value represents the mean ± SD of four replicates.

Previously, we found that an isolate of *C. gloeosporioides* showed an incompatible interaction with the ripe red pepper fruits, but a compatible interaction with the unripe green mature pepper fruits [27]. Typical anthracnose symptoms with necrotic sunken lesions became evident in the infected unripe fruit 3 days after inoculation (DAI) (Figure 1A and 1B). The lesion progressively increased to 25±3.1 mm in diameter at 9 DAI. However, in the infected ripe fruits, necrotic lesions were not observed at 3 DAI, and thereafter, only small spots began to form after 3 DAI, resulting in highly reduced rate of disease development. To determine whether the sesquiterpenoid capsidiol is associated with the incompatible or compatible interactions of the pepper fruits with the anthracnose fungus, we quantified the capsidiol content at the infection sites (Figure 1C). No capsidiol was observed in the absence of fungal inoculation, whereas fungus-inoculated fruits contained varying quantities. The unripe fruit showed only a slight increase of capsidiol, at a concentration of 0.7±0.05 µg/g FW at 1 DAI, which declined thereafter. In contrast, the ripe fruit appeared to accumulate up to 6.8±0.5 µg/g FW at 3 DAI. The capsidiol amount measured was 25-fold higher in the ripe fruit than in the unripe fruit at 3 DAI. These results demonstrate that the levels of capsidiol are negatively related with the size of the lesions in the pathosystems, suggesting that capsidiol is one of the key factors responsible for the incompatible interaction of the ripe fruits with the anthracnose fungus.

### Antifungal activity of capsidiol against the anthracnose fungus

**Figure 2:**
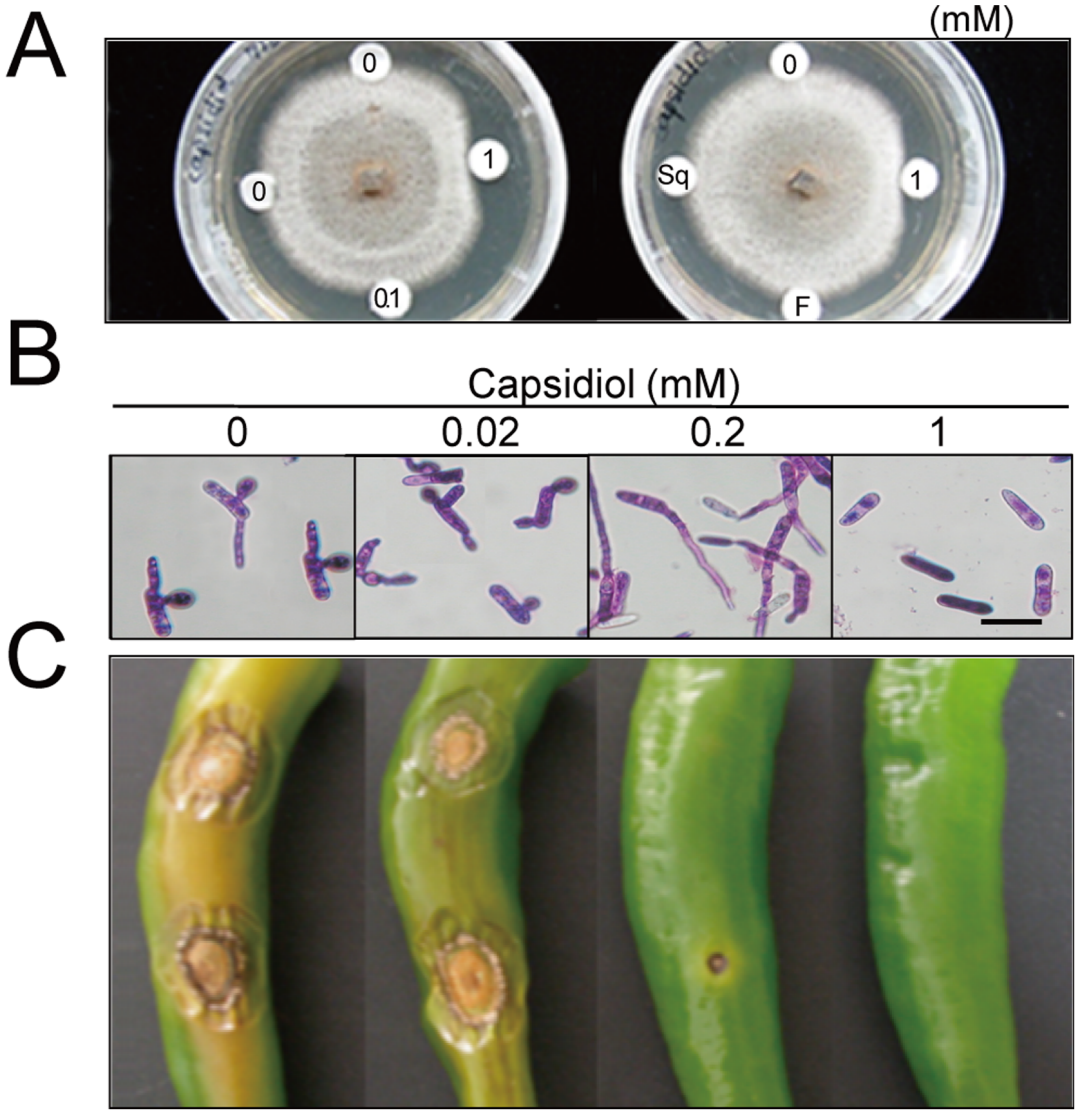
Inhibitory effect of capsidiol on the growth of *C. gleosporioides*. **A**, Anti-fungal activity assay of capsidiol with filter paper discs. Discs were impregnated with 10 µL of 0, 0.1 and 1 mM of capsidiol and placed on the agar media 5 mm apart from the advancing edge of the mycelium. Discs impregnated with 10 µL of 1 mM squalene (Sq) or farnesol (F) were also included as controls. **B**, Evaluation of spore germination and appressorium formation by capsidiol treatment. Spores were amended on cover glasses with 10 µL of capsidiol dissolved in sterile water at concentration of 0, 0.02, 0.2 and 1 mM, then incubated for 24 h. Bar = 10 µm. **C**, Protective activity of capsidiol against anthracnose disease on pepper fruits. 10 µL of 0, 0.02, 0.2 and 1 mM capsidiol was amended with spores of *C. gloeosporioides* on the unripe fruits, and lesion development was observed 9 days after inoculation.

To examine the antifungal activity of capsidiol toward *C. gloeosporioides*, the effects of capsidiol on the growth of the fungus was assessed by a disc assay and microscopic observations. For the disc assay, filter paper discs were impregnated with various amounts of capsidiol and placed on the agar media 5 mm apart from the advancing edge of the mycelium. Radial growth of the fungal colonies was clearly inhibited in response to 1 mM capsidiol (Figure 2A). In contrast, squalene and farnesol (i.e., an acyclic sesquiterpene alcohol) at 1 mM did not affect fungal growth. In addition, microscopic observations revealed that capsidiol had an inhibitory effect on the appressorium formation on the tip of the germination tube of the fungus at 0.2 mM, while spore germination was completely blocked in response to the treatment with 1 mM capsidiol (Figure 2B). Furthermore, we tested whether the capsidiol was able to block anthracnose disease development on pepper fruits inoculated with *C. gloeosporioides*. Results showed that lesion formation was suppressed on the infected fruits by capsidiol treatment in a dosage-dependent manner (Figure 2C). No apparent symptom was observed on the unripe fruit amended with 1 mM capsidiol, whereas the unripe fruit without capsidiol treatment displayed a typical anthracnose symptom, suggesting a protective activity of capsidiol against anthracnose disease. Taken together, these results show that capsidiol has antifungal activity against the anthracnose fungus, *C. gloeosporioides*.

### Expression analysis of genes in the isoprenoid biosynthetic pathway

**Figure 3:**
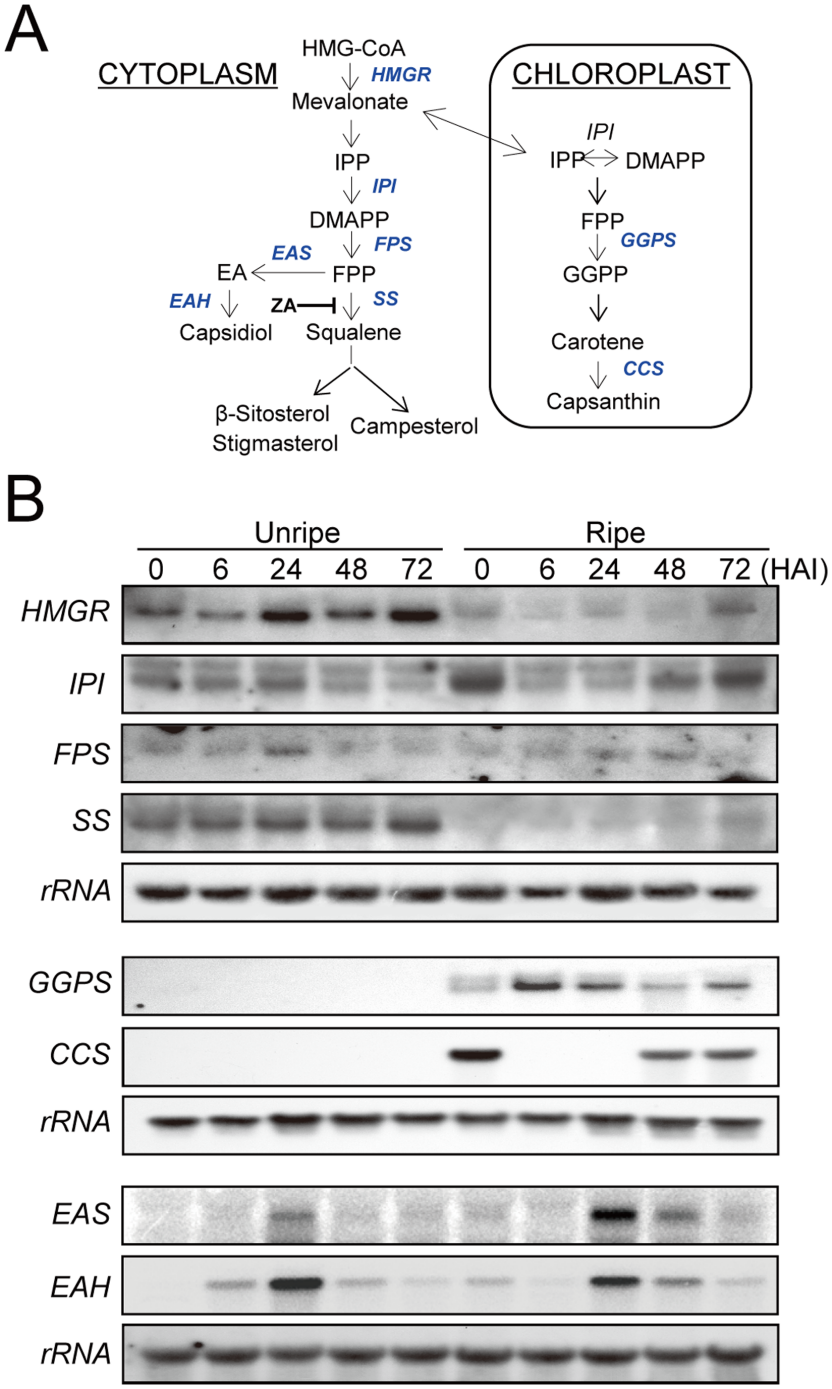
Fungal-induced expression of metabolic pathway genes for phytosterol, sesquiterpene and carotenoid biosyntheses in pepper fruits after inoculation by *C. gloeosporioides*. **A**, Metabolic pathways for phytosterol, sesquiterpene and carotenoid biosyntheses. Genes encoding the enzymes used in the present study are indicated in italics. *HMGR*, 3-hydroxy-3-methyl glutaryl CoA reductase; *IPI*, isopentenyl diphosphate isomerase; *FPS*, farnesyl diphosphate synthase; *SS*, squalene synthase; *GGPS*, geranylgeranyl diphosphate synthase; *CCS*, capsanthin capsorubin synthase; *EAS*, 5-epi-aristolochene synthase (i.e. a sesquiterpene cyclase (*STC*)); *EAH*, 5-epi-aristolochene hydroxylase. ZA (zaragozic acid) is an inhibitor of squalene synthase. **B**, Relative expression of genes involved in phytosterol, sesquiterpene and carotenoid biosynthesis. Total RNA (10 µg/lane) was used for hybridization with ^32^P-labelled cDNA of the indicated gene. The membranes were rehybridized with rRNA to ensure equal loading.

Given the abundant synthesis of capsidiol in the incompatible interaction of the ripe red fruit with the anthracnose fungus and its potent antifungal activity *in vitro*, it is of interest to determine which of the genes in the biosynthetic pathways play a role in the production of capsidiol. To address this issue, we first attempted to clone a subset of selected genes in three different pathways, including the sterol pathway (*HMGR*, *IPI*, *FPS*, and *SS*), the carotenoid pathway (*GGPS* and *CCS*), and the sesquiterpenoid pathway (*EAS* and *EAH*), for use as gene-specific probes (Figure 3A). The core fragments of the above target genes were amplified from RNA of infected ripe fruits at 24 HAI by RT-PCR using degenerate primer sets (Table S1), and the resulting RT-PCR products were cloned and sequenced. The DNA fragments were found to be identical to the corresponding regions of the structural pepper genes encoding *HMGR* (AF110383; *HMGR2*), *FPS* (X84695), *SS* (AF124842), *GGPS* (X80267), *CCS* (X76165) and *STC* (AF061285; *SC1*). Additionally, the sequences of *IPI* and *EAH* were newly obtained (data not shown), exhibiting 91% and 87% identity to the corresponding regions of the nucleotide sequences of tobacco *IPI* (AB049815) and tobacco *EAH* (AF368376).

The expression of genes for sterol and carotenoid biosynthesis in pepper fruits is known to be subjected to developmental control [32]. Thus, the expression of the aforementioned genes in the sterol and carotenoid pathways was examined in pepper fruits infected with anthracnose fungus at two different stages. The results showed that the transcript levels for the genes varied between stages (Figure 3B). Specifically, *HMGR* was expressed constitutively in both types of fruits, but was present in significantly higher levels in the unripe fruit than in the ripe fruit. Upon fungal infection, transient suppression of the *HMGR* transcript was seen at 6 HAI in both fruits, followed by an increase at 24 HAI. Conversely, *IPI* was expressed more abundantly in the ripe fruit than in the unripe fruit. After fungal infection, suppression of *IPI* was much more intense and rapid in the ripe fruits, compared to that in unripe fruits. *FPS* was similar in both fruits, but transient induction by fungal infection was maintained longer in the ripe fruits. *GGPS* and *CCS* were detected only in the ripe fruit, in which the clear induction of *GGPS* was observed upon fungal infection. In contrast, the *CCS* transcript disappeared at 6 HAI until 24 h, but its transcript was recovered during the later stages of the experiment. Capsanthin is known to be a major pepper carotenoid, responsible for the red color, and the exclusive expression of *CCS* in the ripe-fruit was well correlated with the development of red pigment in the ripe-fruit. On the other hand, *SS*, which was developmentally down-regulated in ripe fruits, was gradually increased after fungal infection. More importantly, the penultimate gene *EAS*, involved in sesquiterpene biosynthesis, was not expressed in either type of healthy fruit, but was significantly induced by the fungus in both types of fruits. The induction strength of *EAS* was much stronger in ripe fruits than in unripe fruits, while induction patterns for *EAH* were similar in both fruits.

### Protein levels and enzyme activities of SS and EAS

**Figure 4:**
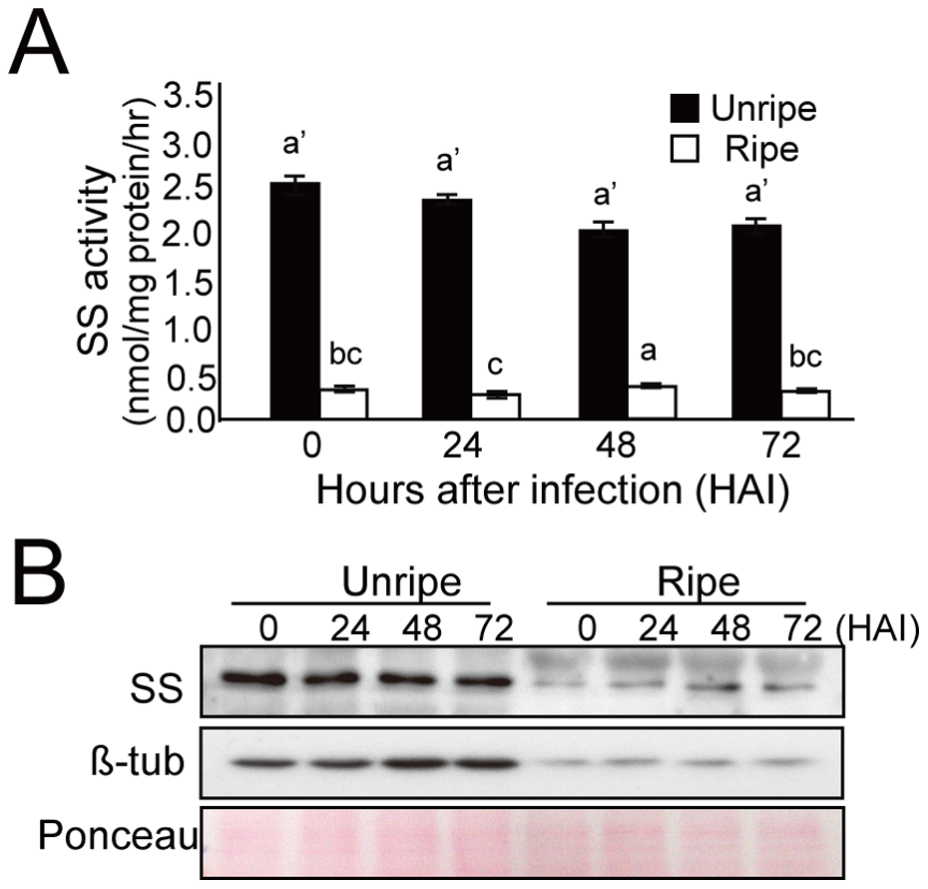
Enzyme activity and protein levels of squalene synthase in unripe and ripe pepper fruits after inoculation by *C. gloeosporioides*. **A**, Enzyme activity assay of squalene synthase (SS). The SS enzyme and [^3^H]-FPP were incubated for 20 min at 30°C. Error bars indicate the standard deviation from three independent measurements. Means with different letters indicate significant difference at *P<0.05*, using LSD and DMRT. **B**, Immunoblot analysis showing the protein levels of squalene synthase. The time after fungal inoculation is indicated in hours on top of each lane. Microsomal proteins (10 µg/lane) were subjected to detect SS protein. β-tubulin and SDS-PAGE of Ponceau S staining were included as loading controls.

**Figure 5:**
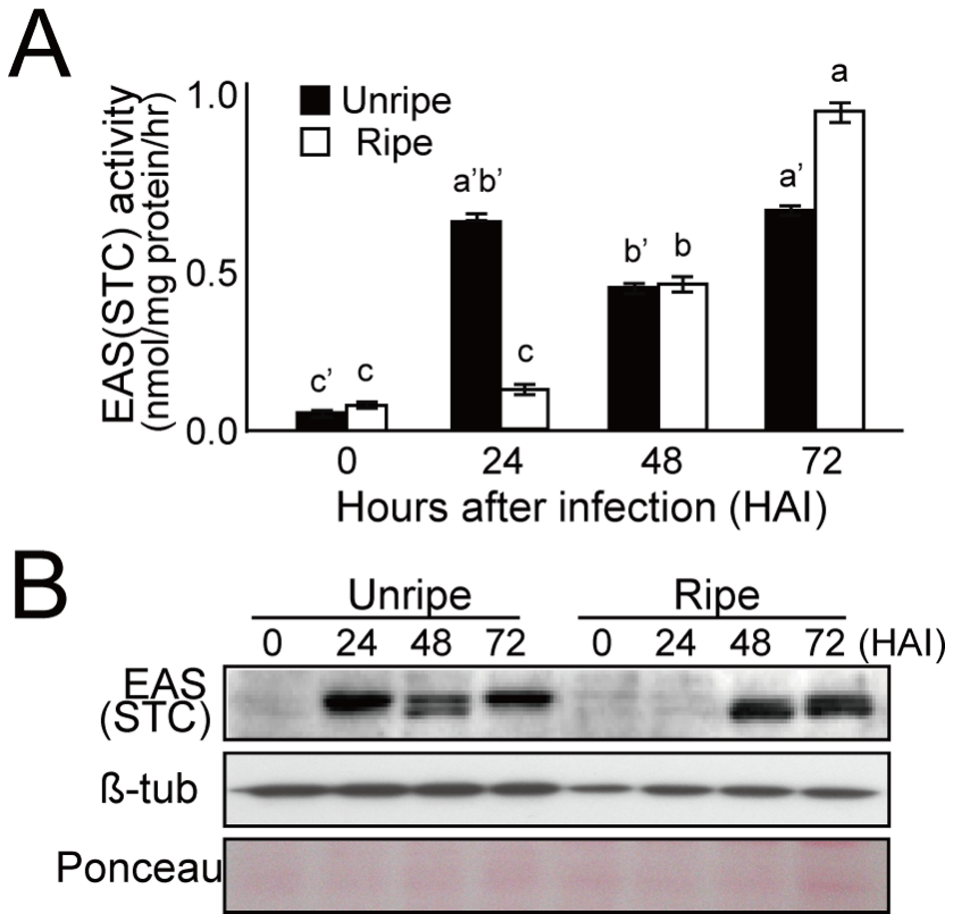
Fungal-induced activity and protein accumulation of 5-epi-aristolochene synthase in pepper fruits after inoculation by *C. gloeosporioides*. **A**, Enzyme activity assay of 5-epi-aristolochene synthase. The EAS/STC activity was measured as described in the Materials and Methods. Error bars indicate the standard deviation from three independent measurements. Means with different letters indicate significant difference at *P<0.05*, using one way ANOVA and DMRT between control and fungal infected fruits. **B**, Immunoblot analysis showing the protein levels of EAS/STC. Soluble proteins (10 µg/lane) were subjected to detect EAS/STC proteins. β-tubulin and SDS-PAGE of Ponceau S staining were included as loading controls.

The expression patterns of SS and EAS were further confirmed by measuring the protein levels and enzyme activities. The SS enzyme, which is primarily responsible for sterol synthesis, was approximately 8 times higher in healthy unripe fruits than in ripe fruits (Figure 4). After fungal infection, the amount and activity of the SS enzyme were slightly decreased in the unripe fruits. In the ripe fruits, a trace amount of SS protein was detected in healthy fruits, and SS protein levels were slightly elevated after fungal infection, along with the enzyme activity. As the counterpart enzyme for sesquiterpene biosynthesis, EAS expression was simultaneously observed in the fruits. In this analysis, a polyclonal antibody against tobacco EAS was used, which did not discriminate EAS from STCs in pepper. The results showed high levels of STC proteins at 24 HAI, when the fungi penetrated the pericarp of the unripe fruit, followed by a decrease at 48 HAI (Figure 5). A second band with a smaller molecular weight was also detected in the unripe fruit at 48 HAI. Conversely, in infected ripe fruits showing capsidiol accumulation, the induction of STC proteins was delayed until 48 HAI, and was maintained at peak levels thereafter. In healthy fruits, STCs were not detectable at both unripe and ripe stages. Taken together, the multiple bands with different mobility of STCs that were detected using polyclonal anti-tobacco EAS antibody in infected fruits may indicate the presence of multiple STC isozymes in the pepper genome. Total STC enzyme activity that was expressed as a concentration of cyclic products from FPP was closely correlated with the levels of the STC proteins in the immunoblot. These results indicate that enzymatic products could be formed over a specified periods in fruits after fungal infection, and also that the reaction products in infected fruits can vary depending on the stage of ripening.

Since the EAS expression analysis by immunoblot showed at least two bands and high EAS activities were observed in the unripe fruits that do not synthesize capsidiol, several STCs might be existed in the pepper. Although a pepper *STC* gene, *SC1* was previously reported to have EAS activity [20], there is still uncertain which STC gene is responsible for the capsidiol production. Thus, we examined the expression of four known *STC* genes (*SC1*, *SC2*, *PSC*1 and *PSC*2) in *C. annuum* to further investigate the *EAS* gene possibly involved in capsidiol biosynthesis. Gene-specific primers were used for RT-PCR and real-time PCR analyses (Table S2). Results showed that the expression of *SC1* and *SC2* was highly induced in both unripe and ripe fruits after fungal infection, but the expression level of *SC1* was much higher than *SC2* (Figure S1). Intense signals of *SC1* were detected in the unripe and ripe fruits at 24 HAI, while prolonged expression of *SC2* occurred in the late period of infection in the ripe fruit (Figure S1B). Additionally, *PSC1* and *PSC2* were exclusively expressed in the ripe fruits, of which the expression level of *PSC2* was diminished over time of the infection period. These analyses demonstrated that the four *STC* genes were induced in different patterns after fungal infection. Based on the expression patterns along with capsidiol accumulation, the *SC1* and/or *SC2* gene was likely involved in capsidiol biosynthesis.

### SS and STC protein levels upon zaragozic acid treatment

**Figure 6:**
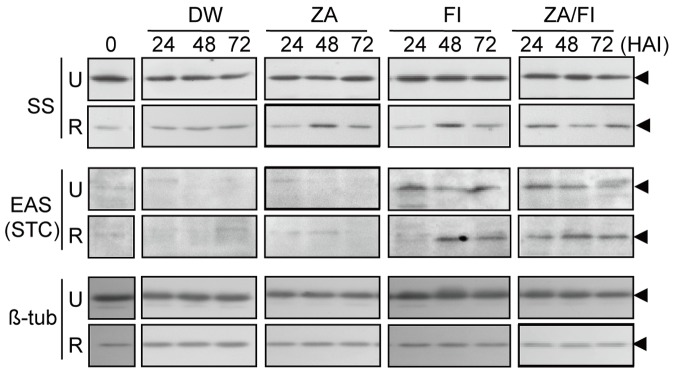
Effects of ZA pretreatment on the fungal-induced expression of SS and EAS proteins in the unripe and ripe pepper fruits infected with *C. gloeosporioides*. Immunoblot analyses were conducted to show the protein levels of SS and EAS. Microsomal and soluble proteins (10 µg/lane) were used for the detection of SS and EAS, respectively. The immunoblots shown are representative of at least three independent experiments. β-tubulin was included as the loading control. The time in hours is indicated on top of the lanes. FI, fungal infection; ZA/FI, FI with pretreatment of 10 µM ZA; ZA, pretreatment of ZA; DW, mock experiments as controls.

To determine if SS activity plays a role in the synthesis of sesquiterpenes, pepper fruits were challenged with an SS inhibitor, zaragozic acid (ZA). After pretreatment with ZA, protein expression patterns of SS and EAS were then monitored in the infected fruits. Results showed that the ZA treatment increased the SS protein levels in the infected ripe fruits, while it lowered the SS protein levels slightly in infected unripe fruits, compared with the control of DW treatment (Figure 6). Likewise, fungal-induced expression of EAS was also stimulated in the ripe fruits by ZA treatment. The enhancement of EAS level was prominent in the ripe fruits, in which fungal-induced expression of EAS (i.e., ZA/FI) was advanced by one day compared to samples without ZA pretreatment (i.e., FI). These results demonstrate that EAS protein expression is stimulated by inhibiting SS activity in infected fruits, which might increase capsidiol levels in the ripe fruits.

### Accumulation of squalene, phytosterols and capsidiol in response to ZA treatment

**Figure 7:**
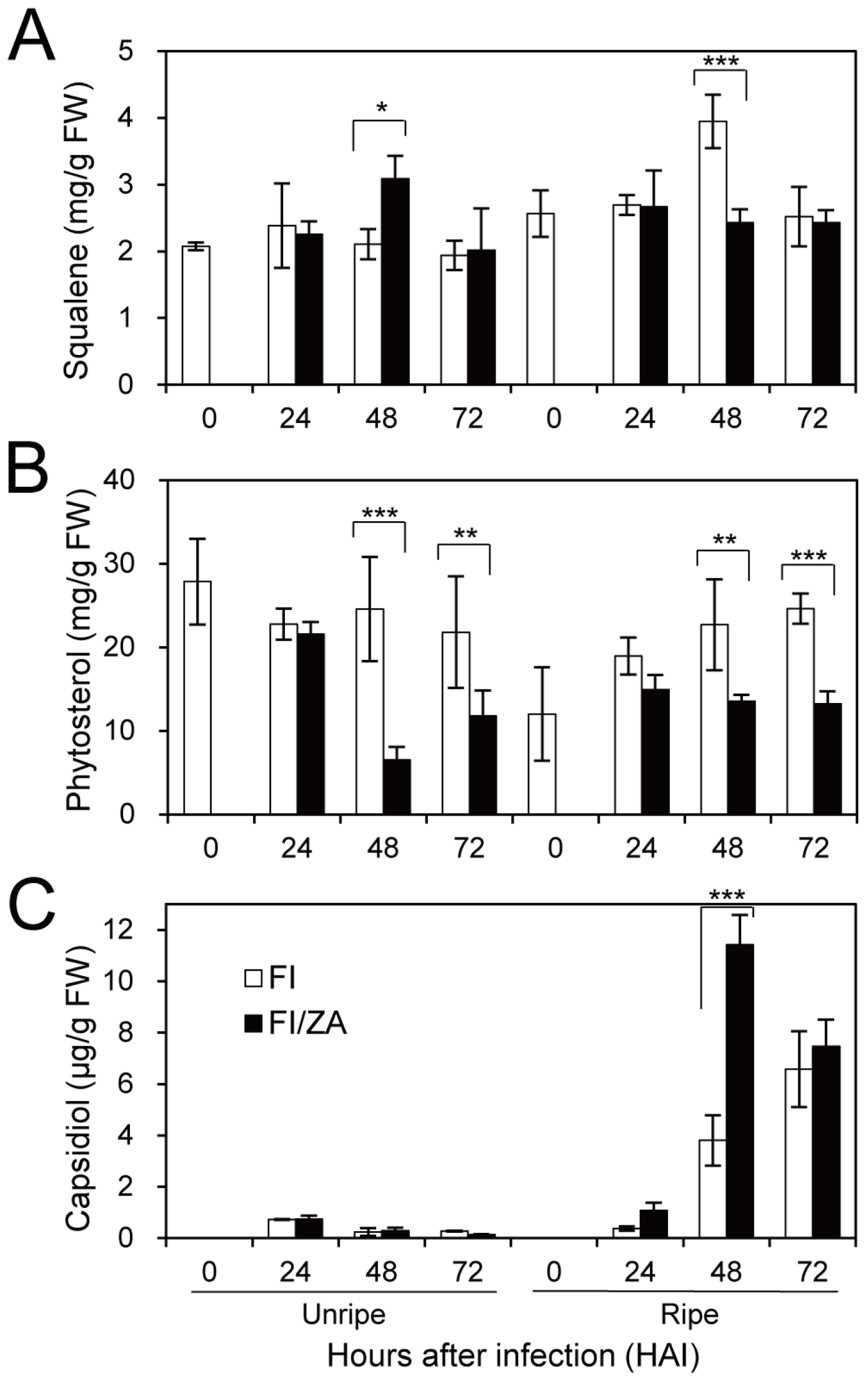
Effects of ZA pretreatment on fungal-induced accumulation of sterols and capsidiol in the unripe and ripe pepper fruits. Squalene (**A**), phytosterols (**B**) and capsidiol (**C**) levels in the infected unripe and ripe pepper fruits, with or without pretreatment of 10 µM ZA, were determined by GC analyses. Unripe/FI and Ripe/FI, unripe or ripe fruits with fungal infection; Unripe/FI+ZA and Ripe/FI+ZA, fungal-infected unripe or ripe fruits with ZA pretreatment. The data represent the mean values ± SD from three independent experiments. Significance test was performed by two-way ANOVA in comparison between ZA pretreated and non-treated infected fruits (*, **, and *** indicating *P*<0.05, 0.01, and 0.001, respectively).

GC analyses were performed to further investigate the changes in the contents of squalene, phytosterols (sitosterol, campesterol and stigmasterol) and capsidiol occurring with ZA treatment during interaction with the anthracnose fungus. The squalene level was slightly higher in the ripe fruits than in unripe fruits in normal condition (Figure 7A). Upon fungal-infection, the level tended to increase in the infected fruits at the early period of infection, before declining. ZA treatment prior to fungal infection led to dwindling squalene in the fruits compared to infected fruits without ZA. In the case of phytosterols, the ripe fruits contained less than half the amount of the unripe fruits. Total amounts of phytosterols were significantly increased upon fungal infection in the ripe fruits, but were reduced in the unripe fruits (Figure 7B). With consecutive ZA treatment and fungal infection, both types of fruit showed severe reduction of phytosterol levels at 48 HAI and 72 HAI, indicating that ZA efficiently suppressed the sterol biosynthesis *in vivo*. On the other hand, the capsidiol contents were almost 2-fold higher in the ripe fruits at 48 HAI following ZA treatment (Figure 7C and Figure S2), indicating that the inhibition of sterol biosynthesis clearly affected the biosynthesis of sesquiterpene. In the unripe fruits, no increase in capsidiol contents was observed with the ZA treatment, even though the contents of phytosterols were reduced. These results represent that metabolic control in pepper fruits take places differentially for common substrate, depending on the stage of ripening. For example, FPP incorporated dominantly into phytosterols in the unripe fruits, while its incorporation into sesquiterpene might be greater in the infected ripe fruit, which developmentally adjusted to reduced carbon allocation to the phytosterols.

## Discussion

Capsidiol, as the major phytoalexin in pepper, accumulated in the infected ripe pepper fruits which showed incompatibility with *C. gloeosporioides*. Fungal-induced responses in the ripe fruits imply that capsidiol plays a functional role in the resistance reactions to the anthracnose fungus (Figure 1 and 2). In the present study, we found that most genes in the isoprenoid pathway, such as *HMGR, FPS*, and *SS*, but not *IPI*, were clearly down-regulated during the ripening stage of pepper fruits (Figure 3B). In contrast, *GGPS* and *CCS* genes were highly expressed in ripe fruits, which explain their massive synthesis of carotenoids such as capsanthin. In addition to differential expression of isoprenoid genes depending upon the fruit stage, fungal infection also caused significant changes in the gene expression patterns related to sterols, sesquiterpenes and carotenoids in the fruits. Most importantly, the expression of the sesquiterpene pathway genes, such as *EAS* and *EAH*, was induced in both unripe and ripe fruits upon pathogen infection. Therefore, the present results provide molecular evidence for the increases in capsidiol levels in ripe fruits infected with the anthracnose fungus, in which up-regulation of the sesquiterpene pathway seems to be closely associated with the enhanced synthesis of capsidiol in response to the fungal pathogen.

In plants, the precursor of isoprenoids IPP is synthesized by two independent pathways, the MVA pathway in the cytoplasm and the MEP pathway in plastids, and the metabolic flows between the two pathways have been known [33]. For sesquiterpene production, the MEP pathway was known to provide IPP for both plastid monoterpene and cytosolic sesquiterpene in snapdragon [26]. In present study, we showed that the expression of *CCS* in chloroplast was transiently blocked in ripe fruits by fungal infection (Figure 3), suggesting a possibility that plastid IPP is available for the synthesis of sesquiterpene when the ripe fruits were challenged with a fungus. However, the contribution of plastid IPP for the sesquiterpene biosynthesis is not clear at this point, so further studies will be necessary to elucidate the cross-flow of IPP between chloroplast and cytosol in the isoprenoid pathway.

The biosynthesis of sesquiterpenes begins with a C_15_ isoprenoid building block (i.e., FPP) and is mediated by the first committed enzyme, EAS. The present study demonstrates that the protein levels and enzyme activities of SS were much higher in healthy unripe fruits than in ripe fruits, which explains the predominant employment of FPP for sterol production in the unripe fruits (Figure 4). In the case of pathogen infection, the expression and enzyme activity of the EAS protein were significantly induced in both unripe and ripe fruits (Figure 5). Therefore, the combination of induction in EAS activity upon pathogen infection and suppression of SS activity during fruit ripening might explain the accumulation of capsidiol observed only in the ripe pepper fruits infected with pathogen. This is consistent with previous results that induction of STC and suppression of SS activities were observed in tobacco cells and potato tubers upon elicitor treatment or fungal infection [21], [34].

During the analysis of EAS activity induced by fungal infection, we found more rapid induction in the unripe fruits than in the ripe fruits (Figure 5). However, the induction pattern fluctuated in the unripe fruits, and two protein bands were observed. Since the polyclonal antibody against tobacco EAS (i.e., an STC) was used for analysis, these results suggest the existence of more than two STC proteins that could be induced by pathogen infection. If we consider the exclusive capsidiol accumulation observed in the ripe fruits 48 h after fungal infection (Figure 1C), the induced protein band in the ripe fruits after 48 HAI might represent an important EAS/STC, observed as the one with the smaller molecules in the immunoblot (Figure 5B). In the case of EAS/STC proteins induced in the unripe fruits, the strong EAS/STC band with higher molecular weight might not be important for capsidiol biosynthesis, because little synthesis of capsidiol was observed in the unripe fruits (Figure 1C). This indicates that the expression of EAS/STC enzymes might not represent the production of capsidiol in the infected pepper fruits, but the induction of authentic EAS among STC enzymes would be critical for the precursor synthesis of capsidiol. These results are consistent with a previous report that strong induction of an EAS in tobacco leaves upon infection with *Ralstonia solanacearum* did not lead to the synthesis of capsidiol [35].

In addition, it has been reported that tobacco leaves inoculated with *Pseudomonas lachrymans* accumulated six different sesquiterpenoid phytoalexins including capsidiol [36], and a fungal sterol, ergosterol, triggered differential accumulation of capsidiol and other bicyclic sesquiterpenoid phytoalexins in tobacco cells [37], which suggests the possibility of functional differences among the different EAS/STC proteins. To identify the *EAS* gene for 5-epi-aristolochene biosynthesis, the expression patterns of four known *STC* genes in pepper fruits were additionally investigated. We found that the *SC1* and *SC2* genes were inducible by fungal infection, while the *PSC1* and *PSC2* genes were not fungal-inducible, but were observed only in the ripe fruits (Figure S1). It is notable that *SC1* was previously reported as a functional EAS [20], and the partial fragment of *SC1* was subcloned using degenerate primers and used for expression analysis in unripe and ripe pepper fruits upon fungal infection by Northern blot (Figure 3B). The results of real-time PCR analyses further showed that *SC1* expression was highly induced by fungal infection (Figure S1B), suggesting its possibility of identity as the functional EAS for capsidiol biosynthesis in pepper fruits. However, these results could not exclude the existence of other EAS proteins for capsidiol biosynthesis. Recently, genome sequencing of hot pepper was completed [38], identifying more STC genes in the genome. Therefore, further functional characterization of the STC genes will be necessary to identify the authentic EAS protein(s) involved in capsidiol production in pepper fruits under fungal induction, which will be also helpful to elucidate the regulation of the sesquiterpene biosynthesis in pepper.

In the cytoplasm, FPP is the common precursor for the biosynthesis of both phytosterols and sesquiterpenes, as the substrate for both the SS and EAS enzymes. To address the role of SS as a branch point enzyme between sterol and sesquiterpene biosynthesis, the SS inhibitor (ZA) was applied to the pepper fruits in the present study. This treatment may lead to the rise of FPP levels in the cytosol, which in turn would increase the substrate availability for EAS. As a result of ZA pretreatment, capsidiol biosynthesis was enhanced in the ripe fruits with fungal infection (i.e., Ripe/FI+ZA in Figure 7C). In the case of phytosterols, synthesis was decreased in the infected fruits by ZA pretreatment (Figure 7B). Consistently, ZA/FI treatments in the ripe fruits induced rapid EAS protein synthesis with higher levels than treatment with FI only (Figure 6). Therefore, our results suggest that the blocking of SS activity with ZA treatment supplies increased amounts of substrate to EAS, resulting in elevated capsidiol biosynthesis upon fungal infection.

Based on the results of the present study, the accumulation of capsidiol in the ripe pepper fruits with fungal infection is mediated by the increased functional EAS levels, which might account in part for the incompatible interaction between ripe pepper fruits and anthracnose fungus. Therefore, EAS, as the key enzyme for capsidiol biosynthesis, may hold an immense potential for biotechnological use in plant disease control. For example, enhanced capsidiol production using the functional EAS under a pathogen-inducible promoter would be useful to confer better resistance against pathogens in plants.

## Supporting Information

Figure S1 **Relative expression of** ***STC*** **genes in unripe and ripe pepper fruits infected with** ***C. gloeosporioides.*** **A**, RT-PCR analysis of *SC1*, *SC2*, *PSC*1 and *PSC*2. **B**, Real-time RT-PCR analysis of *SC1* and *SC2*. Total RNA was extracted from infected fruits at 0, 24, 48, and 72 HAI. Pepper *UBI-3* gene was used for normalization. Error bars indicate the standard deviation from three independent measurements.
(TIF)

Figure S2 **Time-dependent GC profiles of capsidiol extracted from unripe and ripe pepper fruits.** G, green unripe fruit; R, red ripe fruit; F, fungal-infected fruits; FZ, fungal-infected fruit with zaragozic acid treatment. The peppers were inoculated by *C. gloeosporioides* with or without pretreatment with 10 µM zaragozic acid. Dashed lines represent the peaks of capsidiol.
(TIF)

Table S1 **Primers used for cloning of isoprenoid pathway genes.**
(PDF)

Table S2 **Primers used for expression analyses of STC genes.**
(PDF)
