# Supplementary figures and images for: Developmentally Regulated Sesquiterpene Production Confers Resistance to Colletotrichum gloeosporioides in Ripe Pepper Fruits
Source: PLoS One. 2014 Oct 6;9(10):e109453. doi: 10.1371/journal.pone.0109453 (PMC4186859; doi:10.1371/journal.pone.0109453)

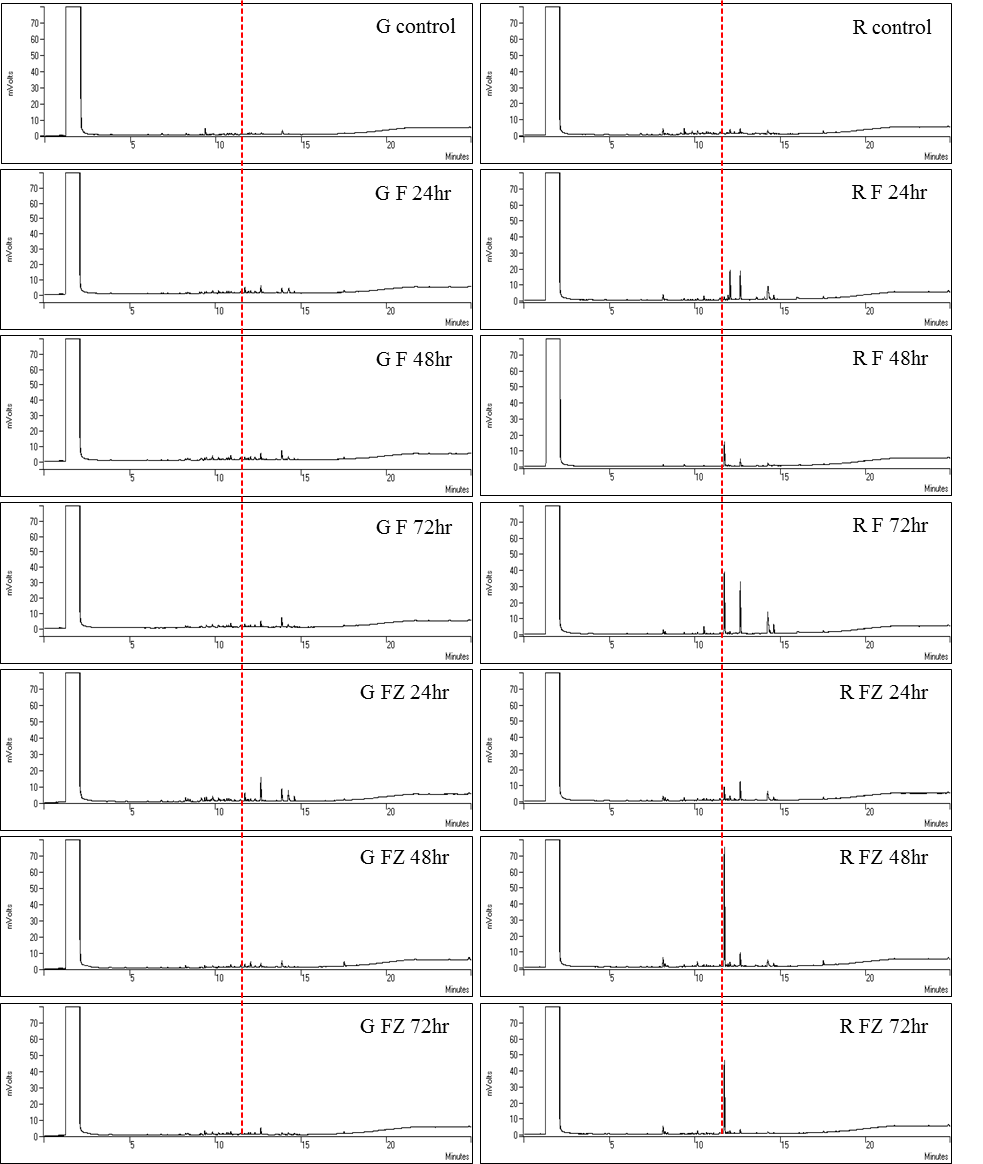

Supplement: Figure S2 — Time-dependent GC profiles of capsidiol extracted from unripe and ripe pepper fruits. G, green unripe fruit; R, red ripe fruit; F, fungal-infected fruits; FZ, fungal-infected fruit with zaragozic acid treatment. The peppers were inoculated by C. gloeosporioides with or without pretreatment with 10 µM zaragozic acid. Dashed lines represent the peaks of capsidiol. (TIF) [file pone.0109453.s002.tif]
