# Supplementary material for: Developmentally Regulated Sesquiterpene Production Confers Resistance to Colletotrichum gloeosporioides in Ripe Pepper Fruits
Source: PLoS One. 2014 Oct 6;9(10):e109453. doi: 10.1371/journal.pone.0109453 (PMC4186859; doi:10.1371/journal.pone.0109453)
Supplement: Table S1 — Primers used for cloning of isoprenoid pathway genes. (PDF) [file pone.0109453.s003.pdf]

Table S1. Primers used for cloning of isoprenoid pathway genes.

Gene	Primers[a]	Size (bp)	Gene name	Accession No.
HMGR	5′-GGTGATGCAATGGGAATGAACATG-3′<br>5′-GTTCCACCACCAACAGTACCAACCT-3′	470	3-Hydroxy-3-methylglutaryl-coenzyme A reductase	AF110383
IPPI	5′-CAAATATAATTGTCATCTGATGGAA-3′<br>5′-CCACCACTTVAWCAAGAAATTGTC-3′	533	Isopentenyl pyrophosphate isomerase	NR[b]
FPPS	5′-CTTGGTTGGTGYATTGAATGGCT-3′<br>5′-CATCCTGAACTTGAAAGTAGRTTCCCA-3′	470	Farnesyl pyrophosphate synthase	X84695
EAS	5′-GCACTCGCTTTCTCCACTACTCA-3′<br>5′-CATTCCTTCATTAATATCCTTCCAT-3′	987	Epi-aristolochene synthase	AF061285
EAH	5′-GARGARYTVAARTACYTAAAGTTRGT-3′<br>5′-GGRYARATCCTCCTTCCACT-3′	284	Epi-aristolochene hydroxylase	NR
SS	5′-GCACTTGACACTGTTGAGGATGATA-3′<br>5′-CTGAAGAAATAAACCCATGGAGTTG-3′	396	Squalene synthase	AF124842
GGPPS	5′-CCCCATGTTATCCATGAAGCAATGCG-3′<br>5′-CGACGTCCTCGCCGTAGATTTTGTGGTT-3′	246	Geranylgeranyl pyrophosphate synthase	X80267
CCS	5′-CAACTCCACTTTTCCAAATCCAACC-3′<br>5′-TCAAACTCATCAACCCAAACACCAT-3′	314	Capsanthin capsorubin synthase	X76165

[a]Degenerate primers: R = A + G, W = A + T, Y = C + T

[b]NR, not reported
