# Supplementary material for: Developmentally Regulated Sesquiterpene Production Confers Resistance to Colletotrichum gloeosporioides in Ripe Pepper Fruits
Source: PLoS One. 2014 Oct 6;9(10):e109453. doi: 10.1371/journal.pone.0109453 (PMC4186859; doi:10.1371/journal.pone.0109453)
Supplement: Table S2 — Primers used for expression analyses of STC genes. (PDF) [file pone.0109453.s004.pdf]

Table S2. Primers used for expression analyses of STC genes.

Gene	Primers	Accession No.
SC1	5′-CCCATACCTATGGAGTTTTTATCT-3′<br>5′-GGCAATGCACTGCACAACTAC-3′	AF061285
SC2	5′-CCTCTTCTGTAACTATGGAGTTTTT-3′<br>5′-GTAGAAACTCCAGACATTCTGAC-3′	AF212433
PSC1	5′-CCGTCTCTACCGAAATCCTTA-3′<br>5′-TATGCATCGCCAGAAGCTCG-3′	AF326118
PSC2	5′-CGACCAACACCTGTCTCTAC-3′<br>5′-GTACTATAGGGAACTTCACTTTG-3′	AF326117
UBQ	5′-TGTCCATCTGCTCTCTGTTG-3′<br>5′-CACCCCAAGCACAATAAGAC-3′	AY486137
